# Genotypic Adaptation of Durum Wheat to Contrasting Water Regimes: Linking Phenology, Chlorophyll Status, Yield Components, and Grain Yield

**DOI:** 10.3390/plants15213448

**Published:** 2026-11-05

**Authors:** Muhammet Öner, Cuma Akıncı

**Affiliations:** 1 Seed Technology Program, Department of Plant and Animal Production, Diyarbakır Agricultural Vocational School, Dicle University, 21280 Sur, Diyarbakır, Türkiye; 2 Department of Field Crops, Faculty of Agriculture, Dicle University, 21280 Sur, Diyarbakır, Türkiye

**Keywords:** durum wheat, drought adaptation, supplementary irrigation, grain yield, SPAD, phenology, stress tolerance index, genotype × environment interaction, semi-arid agriculture

## Abstract

Water scarcity is a recurrent constraint to durum wheat production in semi-arid Mediterranean-type environments, yet genotype performance can change sharply with the timing and severity of seasonal drought. This study quantified phenological, chlorophyll-related, yield-component, and grain-yield responses of 45 durum wheat genotypes grown during 2021–2022 and 2022–2023 under rainfed and supplementary-irrigated field conditions in Diyarbakır, southeastern Türkiye. The experiment comprised equal-sized plots with four replications under each water regime. Supplementary irrigation was delivered by drip irrigation in five applications beginning at stem elongation. Precipitation from sowing to heading was 187.0 mm in 2021–2022 and 285.5 mm in 2022–2023, and April was warmer in the first season (17.9 °C versus 14.7 °C). Grain yield was significantly affected by growing season, water regime, genotype, and all two- and three-way interactions (*p* < 0.01), although diagnostic tests indicated heterogeneous residual variance among environments and the combined fixed-effect ANOVA is therefore interpreted cautiously. Mean grain yield under rainfed conditions was 466.5 and 3254.3 kg ha^−1^ in the first and second seasons, respectively, compared with 4208.1 and 5560.9 kg ha^−1^ under supplementary irrigation. The exceptionally low first-season rainfed yield represents a near-crop-failure environment; stand-count data were not available to separate establishment losses from subsequent drought effects. Pooled drought indices descriptively ranked Fırat-93 highest for STI (0.638) and GMP (3901.4 kg ha^−1^), whereas Şahinbey had the highest pooled YSI (0.518); these index differences were not subjected to inferential significance testing and should not be interpreted as statistically distinct tolerance classes. Grain yield was associated with SPAD and yield components in pooled genotype means, but these associations were environment dependent. The results characterize genotype responses across four site-year × water-regime combinations and provide hypotheses for validation in broader multi-location and multi-year trials.

## 1. Introduction

Durum wheat (*Triticum turgidum* L. subsp. durum) is a tetraploid cereal of exceptional economic and nutritional importance, particularly in Mediterranean, West Asian, North African, and other dryland production systems. Its grain is the principal raw material for pasta, couscous, bulgur, and several traditional foods, while its cultivation has historically been concentrated in environments characterized by cool, wet winters followed by progressively warmer and drier reproductive periods [1,2]. This production geography gives durum wheat an intrinsic association with water-limited agriculture, but it also exposes the crop to increasing climatic instability. Modern production must therefore reconcile high yield potential with resilience to variable rainfall, terminal drought, and episodic heat.

Climate change is intensifying this challenge by altering rainfall distribution, increasing evaporative demand, and raising the probability that water and heat stress coincide during sensitive developmental stages [3,4,5,6,7]. Drought effects are not determined by seasonal precipitation alone. The timing of rainfall relative to stem elongation, heading, anthesis, and grain filling can be more consequential than the seasonal total, because reproductive processes and grain filling are especially vulnerable to water shortage and high temperature [5,6]. In Mediterranean environments, this temporal dimension explains why apparently similar seasonal rainfall totals can generate very different yield outcomes among years.

Durum wheat response to drought is consequently a whole-plant, developmentally regulated process. Water deficit restricts leaf expansion, canopy growth, stomatal conductance, carbon assimilation, nutrient acquisition, spike fertility, and grain filling, but the magnitude of each response depends on genotype and stress intensity [8,9,10,11,12,13]. Genetic variation has been documented for flowering time, plant height, chlorophyll status, root and shoot allocation, thousand-kernel weight, grains per spike, harvest index, and grain yield under water-limited conditions [8,9,10,11,12,13,14,15]. Physiological studies further show that drought tolerance can involve contrasting strategies, including drought escape through earlier development, maintenance of photosynthetic activity, conservative or permissive stomatal regulation, altered nutrient homeostasis, osmotic adjustment, and sustained reproductive sink strength [10,11,12,13].

Phenology is particularly important in Mediterranean-type systems. Earlier heading may reduce exposure of anthesis and grain filling to late-season heat and soil-water depletion, but excessive earliness can also restrict pre-anthesis biomass accumulation. Thus, the value of earliness depends on the target environment and the balance between drought escape and yield-potential formation [5,14,16]. Similarly, SPAD chlorophyll measurements provide a rapid, nondestructive indicator of leaf greenness and can contribute useful information on canopy physiological status, but chlorophyll retention is not synonymous with drought tolerance and should be interpreted together with yield and developmental traits [9,17,18].

The same principle applies to yield components. Grain yield under drought is the integrated result of crop establishment, biomass production, spike formation, grain number, assimilate supply, and grain filling. Studies in durum wheat and other wheats consistently show that water limitation can reduce grains per spike and thousand-kernel weight, although their relative importance changes with the developmental timing of stress [5,6,8,19]. Stem reserve remobilization, harvest index, and reproductive sink maintenance can partially buffer grain filling when current photosynthesis is constrained [19,20,21]. Consequently, breeding decisions based only on final yield may overlook adaptive trait combinations that stabilize performance across variable seasons.

Genotype × environment interaction further complicates selection. Multi-environment studies in durum wheat have repeatedly shown that environment often explains the largest proportion of grain-yield variation, while genotype rankings change across rainfall, irrigation, temperature, location, and season [14,22,23,24,25,26]. These interactions are not simply statistical inconvenience; they represent biologically meaningful differences in adaptation. For drought-prone regions, robust selection therefore requires evaluation across contrasting water regimes and years, ideally supported by traits that help explain why particular genotypes perform well in specific environments.

Yield-based drought indices provide an additional framework for comparing performance under stress and non-stress conditions. MP, GMP, STI, TOL, SSI, and YSI emphasize different dimensions of response: some reward high productivity in both environments, whereas others emphasize proportional yield retention or susceptibility [22,27,28]. Their interpretation must remain cautious because all are mathematical functions of yield under the two water regimes and are therefore not statistically independent of those yields. Combining indices with direct phenological, physiological, and yield-component measurements can nevertheless improve biological interpretation and help distinguish broadly productive genotypes from those expressing stress-specific adaptation.

Recent durum wheat research combines field phenotyping with multivariate and genetic approaches to resolve the multidimensional nature of drought response [12,13,25,29,30,31,32,33]. For the present study, the relevant point is that replicated field phenotyping remains essential because responses observed under controlled conditions do not necessarily translate directly to grain yield under fluctuating soil moisture and temperature. Accordingly, field datasets that combine diverse germplasm, contrasting seasonal water availability, and direct agronomic and physiological measurements remain valuable for describing phenotypic response patterns.

Southeastern Türkiye is an especially informative environment for such evaluation because durum wheat is agronomically important and crops frequently experience irregular rainfall and rising temperature during spring. Previous regional studies have demonstrated strong effects of rainfall, irrigation, genotype, and harvest index on wheat productivity and have emphasized the value of testing germplasm under both rainfed and water-supplemented conditions [34,35,36,37]. Yet, comprehensive field comparisons that integrate phenology, SPAD, yield components, grain yield, and drought indices across contrasting seasons remain limited for locally relevant and breeding germplasm. Recent studies have also addressed post-flowering drought and supplementary irrigation, deficit-irrigation resilience, physiological contributions to yield under water limitation, and multi-environment stability analysis in wheat [38,39,40,41].

Accordingly, this study evaluated 45 durum wheat genotypes during two climatically contrasting growing seasons under rainfed and supplementary-irrigated field conditions in Diyarbakır, southeastern Türkiye. The objectives were to (i) quantify the effects of growing season, water regime, genotype, and their interactions on phenological, physiological, and agronomic traits; (ii) characterize genotype-specific grain-yield responses across four year × water-regime combinations at one site; (iii) compare productivity- and retention-oriented drought indices as descriptive ranking criteria; and (iv) summarize multivariate phenotypic patterns among genotypes. Because only two seasons at one site were available, the study was designed to characterize observed response patterns rather than estimate heritability, genetic correlations, or broad adaptation across a target population of environments.

## 2. Results

### 2.1. Contrasting Climatic Conditions Between Growing Seasons

**Figure 1:**
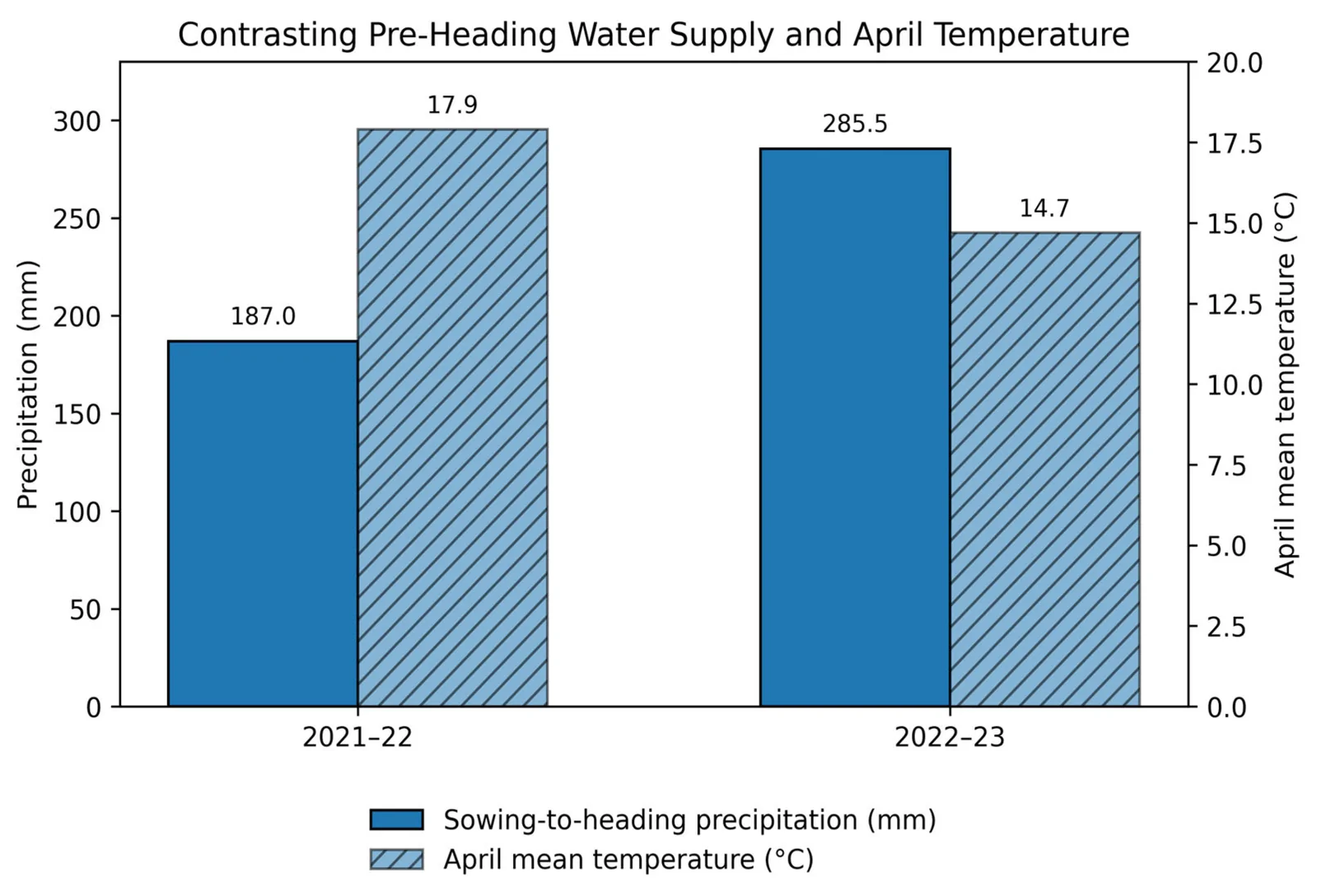
Seasonal climatic contrast between the two field seasons. Paired bars show sowing-to-heading precipitation and April mean temperature for each season; the temperature bars are hatched to distinguish the two variables plotted on separate axes. The figure is intended as a categorical comparison of the two observed seasons; no temporal trend between seasons is implied.

The two seasons had similar total precipitation, but its distribution differed markedly. Total precipitation was 297.6 mm in 2021–2022 and 302.1 mm in 2022–2023, whereas precipitation accumulated from sowing to heading increased from 187.0 to 285.5 mm. The first season was also warmer during April (17.9 °C) than the second season (14.7 °C). These observations document a substantially drier and warmer pre-heading period in 2021–2022 (Figure 1). They do not, by themselves, quantify post-heading crop water status because evapotranspiration and soil-moisture data were unavailable. Month-specific May and June precipitation and temperature data were unavailable in the surviving records; therefore, grain-filling heat and post-heading water supply cannot be quantified at monthly resolution.

### 2.2. Effects of Year, Genotype, and Water Regime

Combined fixed-effect ANOVA detected strong effects of growing season, water regime, genotype, and their interactions (Table 1). For grain yield, all main effects and two- and three-way interactions were significant (*p* < 0.01). However, residual diagnostics showed substantial heterogeneity among the four environments and non-normality for several traits, including grain yield; accordingly, these pooled F-tests are interpreted as descriptive evidence for differences within the observed two-season dataset rather than as inference to a broader population of years. Environment-specific genotype comparisons are therefore emphasized alongside the combined analysis (Supplementary Tables S9 and S10).

A conservative grain-yield sensitivity check using the Y × G mean square as the denominator gave F44,44 = 1.72 (*p* = 0.038), markedly weaker than the residual-error test. Together with the low-to-moderate environment-to-environment rank correlations in Supplementary Table S10, this result reinforces the restricted two-season scope of genotype inference.

### 2.3. Phenological and Physiological Responses

Mean heading time was 150.38 days in 2021–2022 and 152.80 days in 2022–2023. In the first season, heading occurred later under rainfed conditions (151.93 days) than under supplementary irrigation (148.85 days), whereas in the second season heading was 152.12 and 153.48 days, respectively. Thus, the observed response does not support a uniform drought-escape pattern. Year, genotype, water regime, and all interaction terms were significant for heading date, indicating environment-dependent phenological responses among genotypes.

Physiological maturity averaged 185.20 days in 2021–2022 and 190.06 days in 2022–2023. The shorter first-season duration is consistent with stronger seasonal stress at the crop level, but the heading response itself differed by water regime and should not be interpreted as uniformly accelerated development under drought.

Heading-stage SPAD averaged 50.46 in the first season and 52.31 in the second. In 2021–2022, mean SPAD was 49.43 under rainfed conditions and 51.48 under supplementary irrigation; in 2022–2023 the pattern reversed (53.28 and 51.33, respectively). Flowering-stage SPAD was slightly higher under rainfed than supplementary irrigation in both seasons (52.18 vs. 51.98 in 2021–2022 and 52.62 vs. 51.27 in 2022–2023). For flowering-stage SPAD, the year × genotype and water-regime × genotype interactions were significant, but the year × water-regime × genotype interaction was not significant (F = 0.96, *p* > 0.05; Table 1). Thus, flowering-stage SPAD should not be described as responding significantly to every interaction term, and its direction of response to water regime was not uniform across the observed environments.

### 2.4. Growth and Yield Components

Mean plant height increased from 70.92 cm in 2021–2022 to 105.91 cm in 2022–2023. The four environment means were 52.23, 89.61, 101.20, and 110.62 cm for 2021–2022 rainfed, 2021–2022 supplementary irrigation, 2022–2023 rainfed, and 2022–2023 supplementary irrigation, respectively (Table 2).

Mean thousand-kernel weight increased from 38.05 g in 2021–2022 to 46.20 g in 2022–2023. Irrigation increased thousand-kernel weight from 32.61 to 43.48 g in the first season (+10.87 g) and from 34.38 to 58.01 g in the second (+23.63 g); therefore, the absolute irrigation-associated increase was larger in the second season. The 58.01-g environment mean was verified against the replicate-level dataset and the original thesis tables.

Grain weight per spike averaged 0.47 and 1.50 g spike^−1^ under rainfed and supplementary irrigation in 2021–2022 and 1.14 and 1.56 g spike^−1^ in 2022–2023 (Table 2). Grain weight per spike and thousand-kernel weight were measured from different samples (10 sampled spikes versus a separate bulk-grain sample), so algebraic reconstruction of one from grains per spike and the other is not expected.

Spike length averaged 5.87 cm in the first season and 7.35 cm in the second. Spikelet number increased from a seasonal mean of 16.00 to 19.46 spikelets spike^−1^. Environment means were 14.20 and 17.80 under rainfed and supplementary irrigation in 2021–2022 and 19.44 and 19.49 in 2022–2023 (Table 2). Because supplementary irrigation began at stem elongation, after much of spikelet initiation had already occurred, the first-season difference should not be interpreted as a direct causal effect of irrigation on spikelet initiation. It may reflect broader environmental, stand, or spatial differences that cannot be separated with the surviving field records.

### 2.5. Grain Yield Response to Water Regime

Grain yield showed the largest seasonal contrast. The overall seasonal mean increased from 2337.3 kg ha^−1^ in 2021–2022 to 4407.6 kg ha^−1^ in 2022–2023. Rainfed mean yield was only 466.5 kg ha^−1^ in 2021–2022, compared with 3254.3 kg ha^−1^ in 2022–2023; supplementary-irrigated means were 4208.1 and 5560.9 kg ha^−1^. The first-season rainfed mean is therefore best regarded as a near-crop-failure environment rather than a routine drought treatment. Emergence was also markedly later in the first season than in the second, but stand counts were not recorded, so the relative contributions of poor establishment and post-emergence drought to the yield collapse cannot be separated.

Genotype yield rankings differed markedly among environments. Spearman rank correlations were recalculated from all 45 genotype means after matching genotypes by name across the four environment columns in Table 3. The correlations were −0.184 between 2021–2022 rainfed and supplementary irrigation, −0.110 between 2021–2022 rainfed and 2022–2023 rainfed, and approximately 0.000 between 2021–2022 rainfed and 2022–2023 supplementary irrigation. The remaining cross-season correlations were 0.344 between 2021–2022 supplementary irrigation and 2022–2023 rainfed and 0.431 between 2021–2022 supplementary irrigation and 2022–2023 supplementary irrigation. The two 2022–2023 environments were more concordant (*ρ* = 0.680). These rank correlations provide direct evidence of substantial re-ranking, particularly for the near-failure 2021–2022 rainfed environment, rather than relying on interaction significance alone. Complete genotype means are presented in Table 3, verified Tukey HSD groupings in Supplementary Table S9, and the exact rank-correlation matrix in Supplementary Table S10.

### 2.6. Drought-Tolerance Indices and Multivariate Genotype Response

Pooled across seasons, supplementary-irrigated (Yp) and rainfed (Ys) genotype means were used to calculate TOL, MP, GMP, SSI, STI, and YSI (Table 4). Stress intensity differed sharply between seasons (SI = 0.889 in 2021–2022 and 0.415 in 2022–2023). Because the pooled Ys values are strongly influenced by the much higher-yielding 2022–2023 rainfed environment, pooled indices should not be interpreted as summaries of the severe season alone. Moreover, Yp in 2021–2022 was not demonstrably stress-free because irrigation began only at stem elongation.

**Figure 2:**
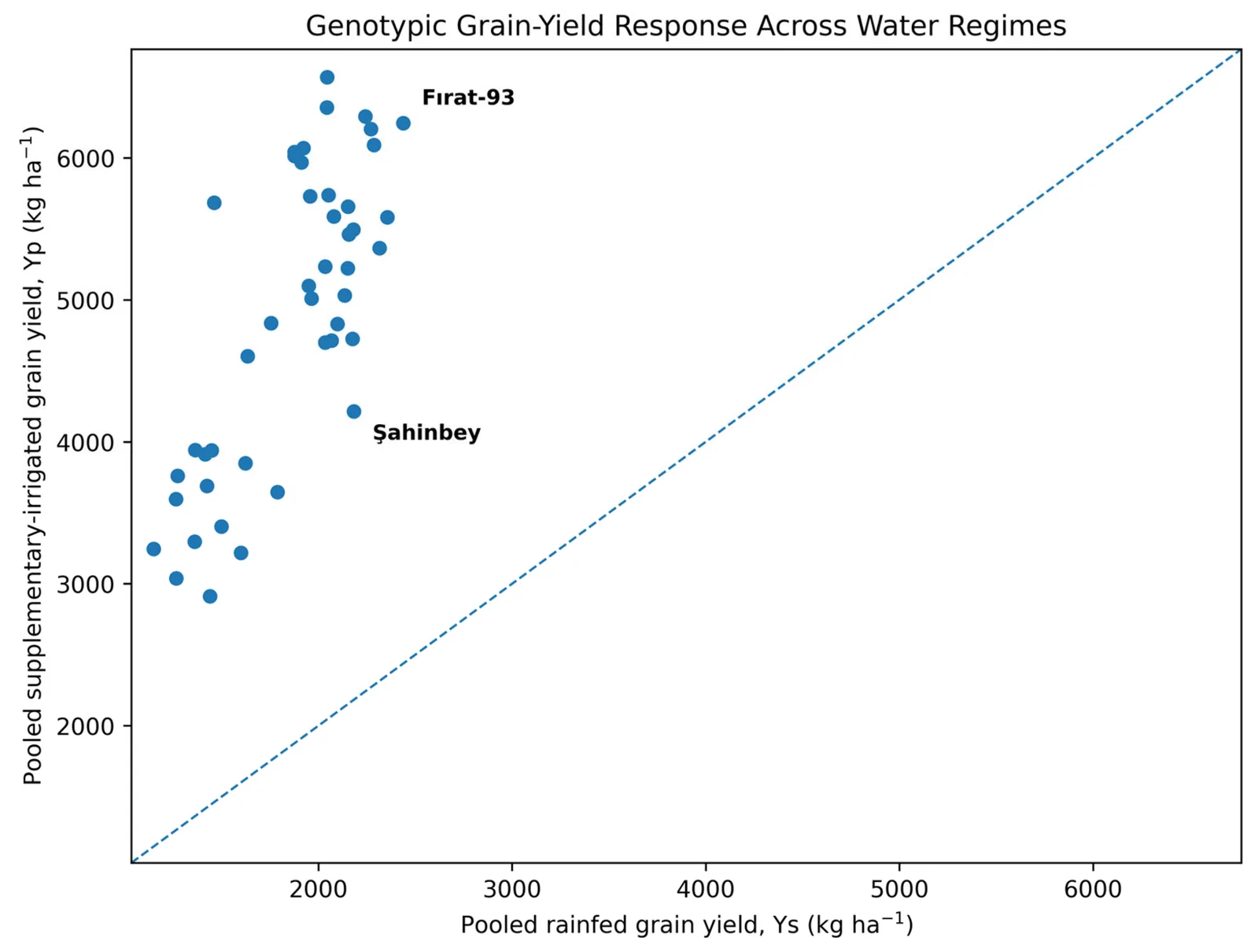
Genotypic grain-yield response under rainfed and supplementary-irrigated conditions. Fırat-93 and Şahinbey are labeled as examples of high pooled STI/GMP and high pooled YSI, respectively. The indices are descriptive treatment-based rankings; the figure does not imply statistically distinct tolerance classes.

The pooled indices provide descriptive, not inferential, rankings. Fırat-93 had the highest pooled STI (0.638) and GMP (3901.4 kg ha^−1^), but Sham-1, Burgos, Sena, and Bağacak had similarly high productivity-oriented rankings; no confidence intervals or significance tests were calculated for differences among index values. Season-specific rankings also differed: in 2021–2022, Bağacak ranked first for STI and GMP, while Eyyubi ranked first for YSI; in 2022–2023, Sena ranked first for STI and GMP, while Gündaş ranked first for YSI (Supplementary Table S4). High pooled YSI values often occurred in genotypes with modest absolute yield because the ratio rewards a smaller proportional reduction. Therefore, YSI is interpreted only alongside an explicit productivity criterion and not as a stand-alone indicator of superior adaptation. The relationship between pooled rainfed and supplementary-irrigated genotype yield is illustrated in Figure 2.

**Figure 3:**
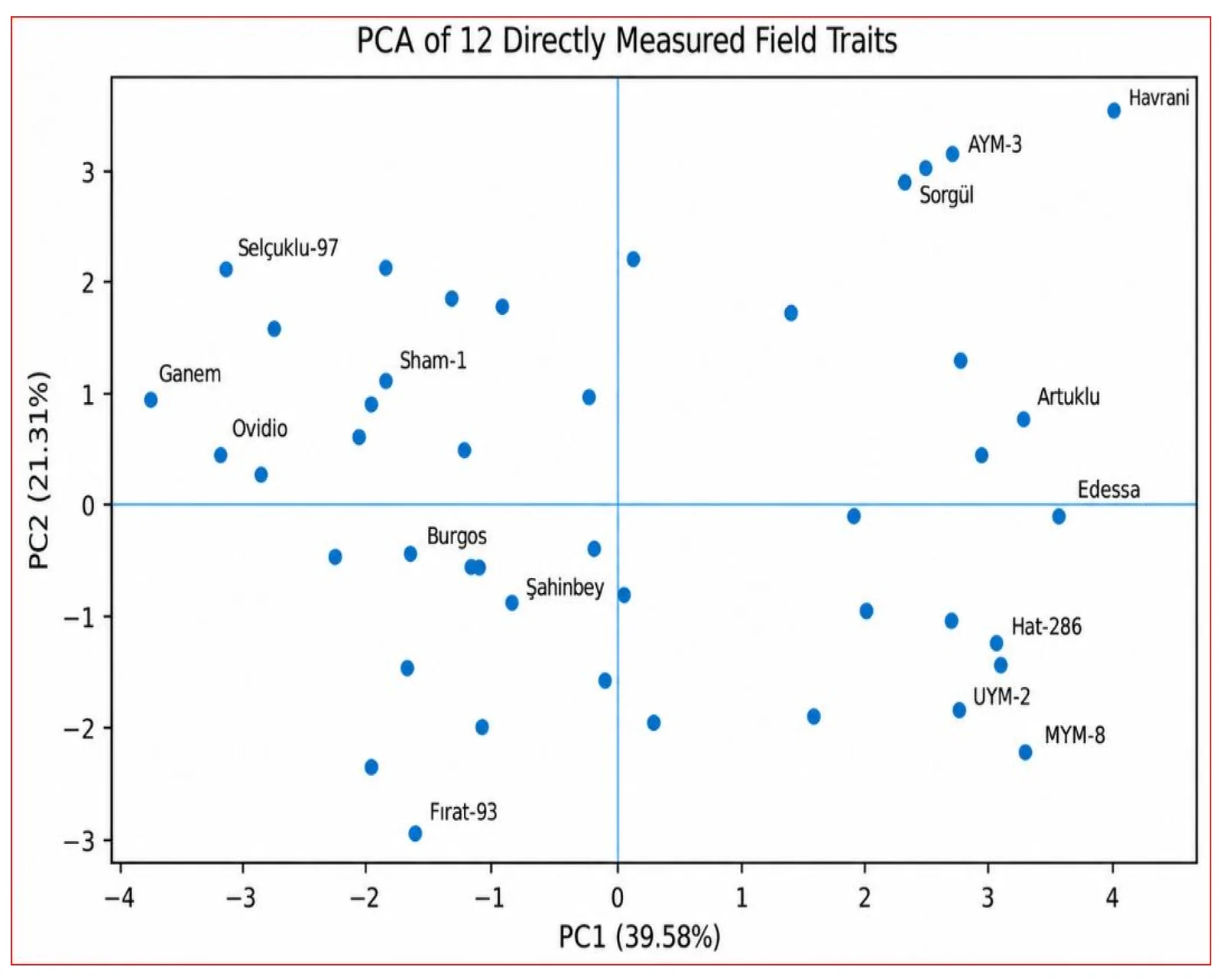
PCA of 45 durum wheat genotypes based on pooled standardized means of 12 directly measured field traits. PC1 and PC2 explained 39.58% and 21.31% of standardized variation, respectively. Full genotype names are shown for selected multivariate extremes to improve legibility; complete scores are provided in Supplementary Table S6.

PCA of pooled genotype means for the 12 directly measured traits showed that PC1 and PC2 explained 39.58% and 21.31% of standardized variation, respectively (60.89% cumulatively; Figure 3; Supplementary Table S6). In the axis orientation reported in Figure 3 and Supplementary Table S6, PC1 contrasted later, taller genotypes (heading time, 0.869; plant height, 0.875; physiological maturity, 0.613) with genotypes having higher SPAD and grain yield (flowering SPAD, −0.804; heading SPAD, −0.774; grain yield, −0.779). PC2 was associated most strongly with spikelets per spike (0.732), grains per spike (0.726), grain weight per spike (0.618), and physiological maturity (0.597). PCA axis signs are mathematically arbitrary: reversing all scores and loadings on an axis would yield an equivalent solution, so interpretation is based on relative directions and magnitudes rather than on the absolute sign itself. The loadings quoted here were rechecked directly against Supplementary Table S6 and the orientation used in Figure 3. Because pooled genotype means were used, these axes summarize overall phenotypic profiles across environments and do not isolate genetic effects from environment-specific responses.

**Figure 4:**
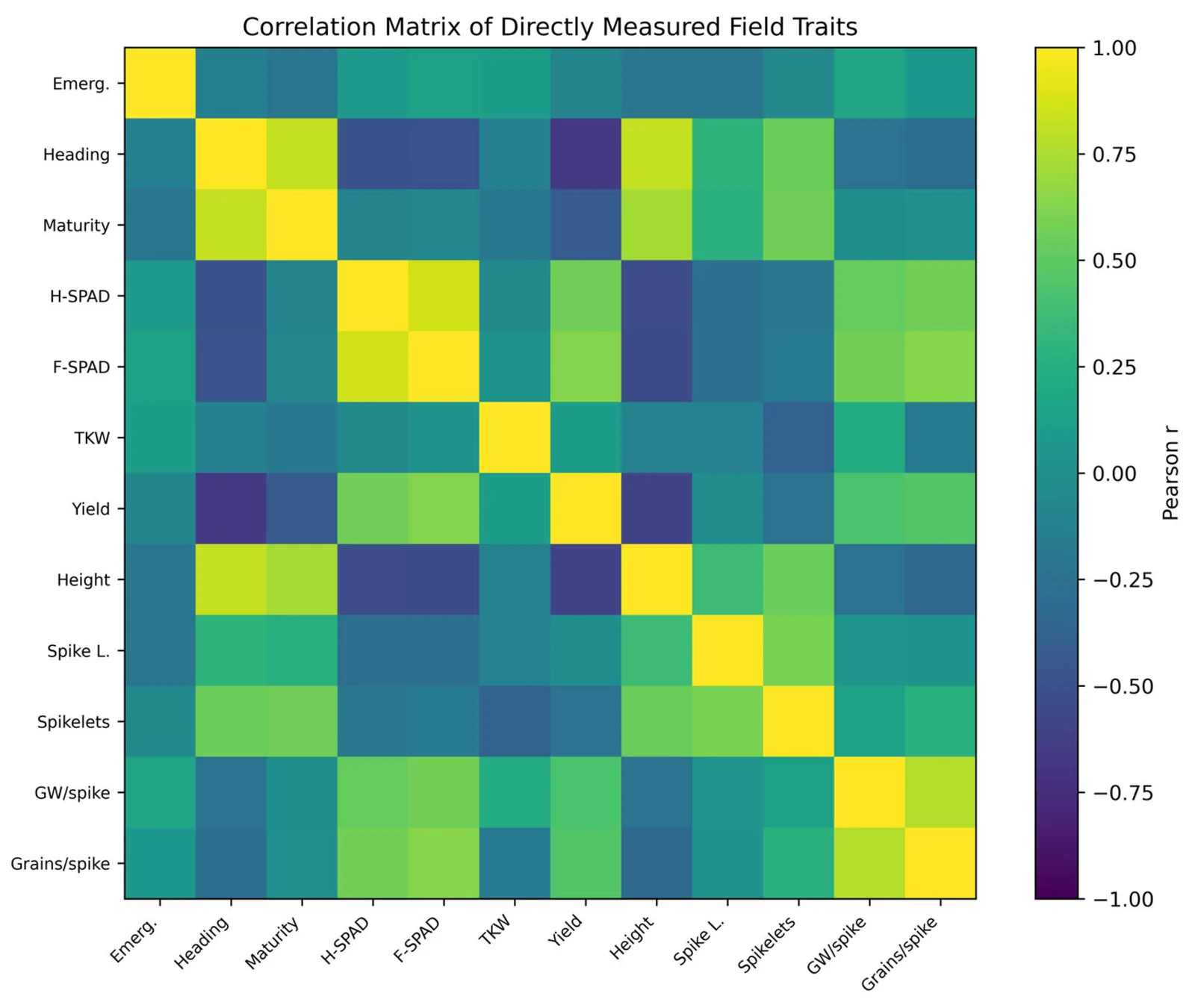
Pearson correlation heatmap for the 12 directly measured field traits calculated from pooled genotype means. Because correlations pool four contrasting environments, they are interpreted as descriptive overall associations; environment-specific sensitivity correlations are provided in Supplementary Table S10.

Correlation patterns were strongly environment dependent (Figure 4; Supplementary Table S10). Across pooled genotype means, where each genotype was first averaged across all four season × water-regime environments, grain yield correlated with flowering-stage SPAD (*r* = 0.631), heading-stage SPAD (*r* = 0.564), grains per spike (*r* = 0.454), grain weight per spike (*r* = 0.433), heading time (*r* = −0.672), plant height (*r* = −0.594), and maturity (*r* = −0.420). However, flowering-SPAD versus yield correlations ranged from −0.050 to 0.791 across individual environments, and heading-time versus yield correlations ranged from −0.137 to −0.740. Therefore, the pooled negative heading-time correlation does not demonstrate a phenological mechanism that operated consistently across water regimes; it is a descriptive association among four-environment genotype averages.

**Figure 5:**
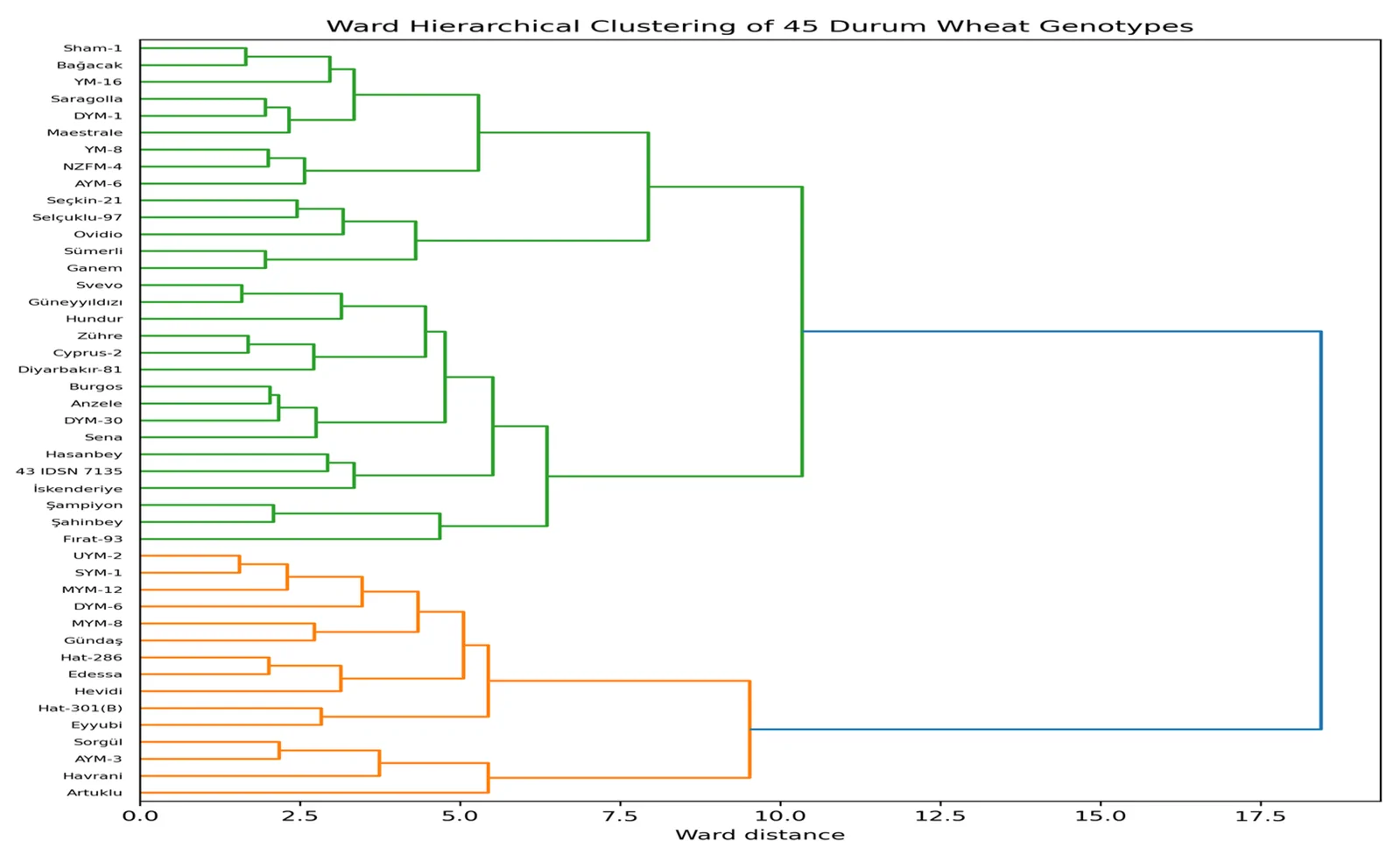
Ward hierarchical clustering of 45 durum wheat genotypes based on pooled standardized means of the 12 directly measured field traits. Full genotype names are shown; cluster membership and cluster-level trait profiles are provided in Supplementary Tables S7 and S10. Green and orange branches indicate the two main genotype clusters, while blue branches represent the higher-level linkage connecting these clusters.

Ward clustering of the same pooled standardized direct-trait matrix was summarized using a four-cluster dendrogram cut for descriptive interpretation (Figure 5; Supplementary Tables S7 and S10). No retained formal optimization criterion for selecting exactly four clusters was available, so the four-cluster solution should not be interpreted as a uniquely supported number of biological groups. Cluster 1 (*n* = 14) combined the highest mean grain yield (3833.9 kg ha^−1^), relatively early heading (149.7 d), and high SPAD; Cluster 3 (*n* = 16) had similar early heading (149.0 d), the highest mean thousand-kernel weight (43.6 g), and high yield (3704.3 kg ha^−1^). Cluster 2 (*n* = 11) had later heading (155.6 d), lower SPAD, and the lowest mean yield (2452.5 kg ha^−1^), whereas Cluster 4 contained only four genotypes and was latest and tallest (157.6 d; 99.2 cm) with intermediate yield. The small size of Cluster 4 limits group-level comparisons with the larger clusters. These clusters are descriptive phenotypic groupings rather than estimates of heritable genetic classes.

## 3. Discussion

### 3.1. Seasonal Contrast and Establishment

The dominant feature of the experiment was the contrast between the two observed seasons. The 2021–2022 rainfed mean yield (466.5 kg ha^−1^) approached crop failure, whereas the 2022–2023 rainfed mean reached 3254.3 kg ha^−1^. Lower sowing-to-heading rainfall and a warmer April in the first season are consistent with strong pre-heading stress, but rainfall totals alone cannot establish terminal drought because post-heading precipitation, evapotranspiration, and stored soil water jointly determine crop water status. The absence of stand counts is also important: delayed emergence in the first season indicates establishment stress, but the dataset cannot partition yield loss between establishment failure and later drought. Comparable Mediterranean studies likewise show that rainfall timing and season strongly modify wheat performance [5,14,22,23,38]. A controlled Dicle University study in bread wheat likewise documented strong genotype-dependent performance losses under imposed water deficit, although its pot-based design differs from the present field experiment [42].

Supplementary irrigation produced a very large yield difference in 2021–2022 and a smaller difference in 2022–2023, but the first-season irrigated treatment had already experienced water limitation before irrigation began. The study therefore compares two management environments rather than a strictly stress-free potential-yield treatment versus a stress treatment. This distinction is carried through the interpretation of drought indices and prevents overstatement of treatment physiology [23,39].

### 3.2. Phenology and Grain Yield

Across pooled genotype means, earlier heading was associated with higher yield, but the relationship was not consistent enough to define phenology as a universal drought-adaptation axis. In 2021–2022, rainfed plots headed later than supplementary-irrigated plots (151.93 vs. 148.85 d), which is opposite to a simple drought-escape expectation. Environment-specific heading–yield correlations ranged from weak (*r* = −0.137 and −0.085 in the two 2021–2022 environments) to strong negative associations in 2022–2023 (*r* = −0.740 and −0.675). Earlier heading may be beneficial in Mediterranean systems [5,14,16], but the present data support an environment-dependent association rather than a general causal drought-escape mechanism. Recent two-season field work under contrasting sowing and water conditions similarly showed that phenological adaptation to warmer conditions can alter yield formation, reinforcing the need for environment-specific interpretation of heading responses [43].

The interpretation is also constrained by the two-season, single-site design. No heritability, genetic variance, or predicted selection response was estimated, so heading time should be treated as a phenotypic descriptor associated with yield in some environments, not as a validated breeding criterion from this dataset alone.

### 3.3. Relationship of SPAD Values to Grain Yield

SPAD responses were not directionally consistent with irrigation. Flowering SPAD was slightly higher under rainfed than supplementary irrigation in both seasons, while heading SPAD changed direction between years. Although pooled genotype means showed positive SPAD–yield correlations, environment-specific flowering-SPAD correlations ranged from −0.050 to 0.791. The pooled relationship therefore does not demonstrate chlorophyll conservation, delayed senescence, or drought tolerance within each water regime. It simply indicates that, when genotypes were averaged across environments, those with higher SPAD tended to have higher overall yield.

This more limited interpretation is consistent with the role of SPAD as an indicator of leaf greenness influenced by developmental stage, nitrogen status, and stress history [9,17,18]. References [17,27] were rechecked in the context of the revised text, and claims linking SPAD directly to drought tolerance were removed. SPAD may remain useful as a complementary phenotypic measurement, but its predictive value requires validation within target environments rather than inference from pooled correlations alone. Semi-controlled durum-wheat screening has also reported concurrent water-stress effects on heading, SPAD, grain number, and grain weight, supporting the use of these traits as complementary descriptors rather than stand-alone evidence of field drought tolerance [44].

### 3.4. Yield Components and Grain Formation

The first-season rainfed environment reduced grains per spike, grain weight per spike, thousand-kernel weight, plant height, and final yield. These traits describe different components of crop performance, but their correlations with grain yield should not be interpreted as independent causal evidence because grains per spike and grain weight per spike are themselves yield components. The larger absolute irrigation-associated increase in thousand-kernel weight occurred in 2022–2023 (+23.63 g) rather than 2021–2022 (+10.87 g). Grain-weight-per-spike and thousand-kernel-weight values need not reconstruct one another because they were obtained from different samples. The observed spikelet-number difference in 2021–2022 is interpreted only tentatively: because supplementary irrigation began at stem elongation and the original spatial field map was not preserved, the available records cannot separate the relative contributions of treatment timing, spatial heterogeneity, stand differences, or other environmental factors. No causal attribution of spikelet-number differences to supplementary irrigation is therefore made.

Spikelet number was higher in the first-season supplementary-irrigated plots than in rainfed plots, but irrigation began at stem elongation, when much of spikelet initiation had already occurred. Accordingly, the difference cannot be attributed confidently to a direct treatment effect on spikelet initiation and may reflect wider environmental or spatial differences. This is one reason the revised manuscript avoids using yield-component associations as proof of reproductive sink mechanisms or as validated selection criteria [6,19,20,21].

### 3.5. Environment-Dependent Genotype Ranking

The revised rank analysis provides direct evidence of genotype re-ranking. Yield-rank correlations involving the 2021–2022 rainfed environment were weak or negative (*ρ* = −0.184 to approximately 0.000), whereas the two 2022–2023 environments were more concordant (*ρ* = 0.680). Thus, the statement that rankings changed is supported by specific rank comparisons rather than by interaction significance alone. The particularly weak concordance of the near-failure environment also cautions against extrapolating its ranking as a general drought-tolerance hierarchy [22,23,24,25,26,38].

For breeding interpretation, these results identify genotypes that merit further testing rather than establish broad adaptation. Fırat-93, Bağacak, and Sham-1 had high overall means, while Eyyubi, Ganem, and Hevidi were comparatively prominent in the severe first-season rainfed environment. These contrasts should be validated across additional locations and seasons before parental value or stability is inferred [34,35,36,37]. Recent multi-environment evaluation of durum wheat in southern Peru likewise emphasized genotype re-ranking and the value of explicit stability analyses across environments, supporting the need for broader validation before inferring adaptation [45].

### 3.6. Drought Indices as Descriptive Ranking Tools

The drought indices separated productivity-oriented and ratio-oriented rankings, but several limitations are critical. First, pooled indices combine a severe season (SI = 0.889) with a much milder one (SI = 0.415), so pooled Ys values are dominated by the higher-yielding 2022–2023 rainfed environment. Second, the supplementary-irrigated 2021–2022 treatment was not stress free before irrigation began, so Yp is not a true physiological potential yield. Third, index values are deterministic functions of Yp and Ys, and differences among genotypes were not subjected to inferential tests. Accordingly, Fırat-93’s highest pooled STI/GMP and Şahinbey’s highest pooled YSI are presented as descriptive rankings, not statistically distinct tolerance classes [15,22,27,28].

Season-specific results further illustrate why pooled rankings require caution. Bağacak led STI/GMP in 2021–2022, whereas Sena led these indices in 2022–2023. Eyyubi had the highest YSI in the severe season and Gündaş in the milder season. Moreover, high YSI can occur with modest absolute productivity because the ratio rewards a small proportional reduction from a low Yp. Therefore, YSI is informative only when considered together with a minimum productivity criterion and absolute yield.

### 3.7. Multivariate Phenotypic Structure

PCA and Ward clustering summarize pooled phenotypic structure rather than environment-independent genetic structure. In the reported PCA orientation, PC1 primarily contrasted later/taller genotypes with higher-SPAD/higher-yield genotypes, whereas PC2 emphasized spikelet number, grains per spike, grain weight per spike, and maturity; the sign of either PCA axis could be reversed without changing this interpretation. Cluster profiles were consistent with those loadings: Cluster 1 combined high yield, earlier heading, and high SPAD; Cluster 3 combined early heading, high thousand-kernel weight, and high yield; Cluster 2 was later, lower-SPAD, and lower yielding; and Cluster 4 was the latest and tallest group. The four-cluster dendrogram cut was used descriptively rather than selected by a retained formal optimization criterion, and Cluster 4 contained only four genotypes, so comparisons involving that group are necessarily less robust than comparisons among the larger clusters. These descriptive groupings help organize phenotypic diversity but do not substitute for heritability, genetic-correlation, AMMI/GGE, or multi-location stability analyses. Complementary field-phenotyping approaches based on chlorophyll fluorescence and vegetation indices have also been demonstrated in durum wheat and could extend future physiological characterization beyond SPAD alone [46].

Because the PCA and clustering used means pooled across four contrasting environments, part of the multivariate pattern can reflect environment-specific responses. The revised manuscript therefore avoids treating clusters as genetic adaptation classes. Future multi-environment trials should use environment-specific or mixed-model genotype predictions and stability analyses to distinguish repeatable genetic structure from seasonal environmental effects.

### 3.8. Scope, Limitations, and Future Research

The present study is best viewed as a detailed two-season field characterization at one site. It identifies phenotypic contrasts and candidate genotypes for further testing, but it does not estimate heritability, genetic correlations, response to selection, or broad adaptation. The unusually low 2021–2022 rainfed yield, missing stand counts, unavailable irrigation depth, incomplete spatial randomization records, and detected residual heterogeneity further limit causal interpretation. These limitations have been made explicit so that agronomic associations are not presented as proven physiological mechanisms or validated breeding criteria.

Future work should evaluate the candidate genotypes across multiple locations and at least several additional seasons using fully documented water application, plot-level soil-moisture or water-balance measurements, stand counts, and mixed-model multi-environment analyses. AMMI/GGE or related stability methods could then quantify repeatable genotype ranking and crossover. Additional physiological measurements such as canopy temperature, root traits, carbon-isotope discrimination, and senescence dynamics could test mechanisms suggested by the present phenotypic associations. Recent rainfed-wheat work integrating a multi-trait genotype–ideotype distance index with explainable machine learning illustrates a complementary route for future multi-trait selection once broader, well-structured training environments are available [47].

The three-year interval between the 2022–2023 field season and submission reflects completion of the doctoral research, consolidation and verification of the full replicate-level dataset, and preparation of the publication analysis. No additional field seasons were added after the original two-season experimental plan. This interval does not expand the environmental scope of the dataset, and the revised manuscript therefore states the two-season limitation explicitly.

Taken together, the results support cautious use of complementary phenotypic evidence rather than a single label of drought tolerance. High absolute productivity, proportional yield retention, phenology, SPAD, and yield components describe different aspects of performance, and their relevance must be validated in the target environments for which selection decisions will be made.

## 4. Materials and Methods

### 4.1. Plant Material

The field experiment comprised 45 durum wheat genotypes representing commercial cultivars, locally collected germplasm from southeastern Türkiye, breeding lines developed at Dicle University, and introduced material. The complete genotype identification and source/origin information are provided in Supplementary Table S1.

### 4.2. Experimental Site, Soil, and Climatic Conditions

The experiment was conducted at the Dicle University Faculty of Agriculture field-crops research area in Diyarbakır, southeastern Türkiye, during the 2021–2022 and 2022–2023 growing seasons. The region has a continental semi-arid climate characterized by hot, dry summers and cold, relatively wet winters. Long-term annual precipitation is approximately 496 mm. The two seasons constitute the complete field duration of this doctoral experiment; broader temporal inference therefore requires additional years and locations.

In 2021–2022, mean temperature, total precipitation, and mean relative humidity during the experimental period were 12.3 °C, 297.6 mm, and 60.00%, respectively. Corresponding values in 2022–2023 were 12.8 °C, 302.1 mm, and 62.57%. Precipitation from sowing to heading was 187.0 mm in the first season and 285.5 mm in the second season, both below the long-term value of 342.1 mm. April mean temperature was 17.9 °C in 2021–2022 and 14.7 °C in 2022–2023. These data document a substantially drier and warmer pre-heading period in the first season. Month-specific May and June precipitation and temperature records were not retained in the surviving experimental dataset and therefore cannot be reported or reconstructed for this revision. Subtracting sowing-to-heading precipitation from seasonal totals suggests that more rain fell after heading in the first season than in the second; however, evapotranspiration, soil-water storage, and plot-level soil-moisture data were not recorded, so post-heading water balance and terminal-drought intensity cannot be quantified from rainfall totals alone.

The soil was clayey in both seasons. In 2021–2022, soil pH was 8.12, lime content 11.56%, organic matter 0.96%, available phosphorus 15.4 kg ha^−1^, and potassium 862.8 kg ha^−1^. In 2022–2023, soil pH was 7.47, lime content 9.8%, organic matter 1.05%, available phosphorus 14.8 kg ha^−1^, and potassium 882.0 kg ha^−1^. These between-season soil differences are reported explicitly because year effects cannot be separated completely from field/soil differences in a two-season field study. The surviving records do not provide plot-level soil-moisture measurements or a sufficiently detailed previous-crop record for inclusion in the analysis.

### 4.3. Experimental Design and Crop Management

The field experiment was conducted during two consecutive growing seasons under rainfed and supplementary-irrigated conditions with four replications. Forty-five durum wheat genotypes were evaluated under both water regimes. All plots had identical dimensions. The surviving dataset identifies four replication labels (1–4) within each season and records season, water regime, and genotype for every observation; however, it does not preserve a field map showing the physical position of those replications. Consequently, whether rainfed and supplementary-irrigated plots were spatially interspersed within replications or separated into irrigation strips/blocks cannot be established from the surviving records. The original spatial randomization map, assignment of water regimes to irrigation blocks, and any documented main-plot/subplot hierarchy are likewise unavailable. A split-plot error structure therefore cannot be reconstructed reliably, and imposing one retrospectively would require assumptions not supported by the records. Each plot consisted of two 3-m-long rows spaced 0.20 m apart. Sowing was performed using a two-row experimental plot seeder on 7 December 2021 and 8 December 2022 at a target density of 500 seeds m^−2^. Border-row configuration, net harvested area, and lateral water movement between rainfed and irrigated plots were not documented. The complete dataset comprised 720 observations (45 genotypes × 2 water regimes × 2 seasons × 4 replications).

Weed control combined mechanical removal with a broadleaf herbicide containing 2,4-D 2-ethylhexyl ester and 6.25 g L^−1^ florasulam. The surviving records do not provide a verified 2,4-D concentration or herbicide application rate; these values are therefore not reconstructed. At sowing, the records report applications equivalent to 60 kg ha^−1^ N and 60 kg ha^−1^ P; the phosphorus fertilizer form (elemental P versus P2O5-equivalent notation) is not recoverable from the surviving records and is reported here exactly as recorded. An additional 60 kg ha^−1^ N was applied at stem elongation on 5 March 2022 and 9 March 2023.

### 4.4. Water Regimes

Two water regimes were evaluated: rainfed and supplementary irrigation. Supplementary irrigation was supplied through a drip-irrigation system beginning at stem elongation and repeated at 15-day intervals for a total of five applications. The surviving records document the timing and number of irrigation events but not a verified water depth or volume per event; irrigation amount, evapotranspiration replacement, and water-use efficiency therefore cannot be reconstructed. Importantly, the supplementary-irrigated treatment should not be interpreted as a fully non-stressed potential-yield environment: in 2021–2022, marked rainfall limitation occurred before the first irrigation, exposing both treatments to early-season water limitation. Consequently, the symbols Yp and Ys used in classical drought-index equations denote the supplementary-irrigated and rainfed treatment means in this experiment, respectively, rather than proven stress-free and stress environments.

### 4.5. Phenological, Physiological, and Agronomic Measurements

Days to emergence were recorded when approximately 50% of coleoptiles had emerged above the soil surface. Days to heading were calculated from sowing until approximately 70% of plants in a plot had the spike emerged halfway from the flag-leaf sheath, and physiological maturity was recorded when approximately 95% of plants had yellowed. Flag-leaf chlorophyll status was measured at heading and flowering between 10:00 and 12:00 h using a SPAD-502 Chlorophyll Meter. The surviving field protocol confirms the two measurement stages and time window but does not specify the number of leaves or repeated SPAD readings per plot; no number is reconstructed here. Before harvest, plant height was measured on 10 randomly selected headed plants from each row. Spike length, spikelet number, grain number, and grain weight per spike were determined from 10 randomly sampled spikes per plot. Thousand-kernel weight was calculated from a separate bulk-grain sample by weighing 400 kernels and multiplying by 2.5. Thus, grains per spike × thousand-kernel weight/1000 is not expected to equal grain weight per spike because the measurements came from different biological samples and sampling units. Grain harvested from each plot was weighed with 0.01 g precision and converted to kg ha^−1^ for reporting (1 kg da^−1^ = 10 kg ha^−1^).

### 4.6. Statistical Analysis

Field data were analyzed with a balanced fixed-effect factorial model containing growing season (Y), water regime (W), genotype (G), and the Y × W, Y × G, W × G, and Y × W × G interactions, with replication retained within season. Because only two seasons were observed, year was treated as the two specific environmental contrasts sampled in this study rather than as a random effect representing a broader population of years; with only two year levels, estimation of a year-variance component would be unstable and would not support robust random-year inference. The original spatial randomization map and water-regime block allocation were not preserved; consequently, a valid split-plot/main-plot error stratum cannot be reconstructed from the surviving records. Type II sums of squares were used for the balanced analysis. The original doctoral analyses were conducted in JMP 17 (SAS Institute Inc., Cary, NC, USA), and all numerical results reported in this article were independently recalculated from the complete 720-observation dataset in Python 3.13 using SciPy 1.17, statsmodels, and scikit-learn 1.8. Residual diagnostics included Shapiro–Wilk tests and Brown–Forsythe tests across the four season × water-regime environments. Several traits, including grain yield, showed departures from normality and/or homogeneous variance; for grain yield, Shapiro–Wilk W = 0.892 (*p* < 0.001) and Brown–Forsythe F = 122.83 (*p* < 0.001). Therefore, the combined ANOVA is presented as a fixed-environment descriptive analysis and is interpreted cautiously, with environment-specific means and within-environment Tukey HSD comparisons emphasized. The complete diagnostic summary is provided in Supplementary Table S10. Genotype comparisons were conducted separately within each season × water-regime environment using the four replicate observations per genotype and the residual variance estimated from that environment-specific genotype model; no common residual error term pooled across the four environments was used for these Tukey HSD tests (α = 0.05). Compact-letter groupings are provided in Supplementary Table S9 and were rechecked against all pairwise Tukey decisions. As a conservative sensitivity check for grain yield, genotype MS was divided by the Y × G MS (rather than by the pooled residual MS), providing an approximate two-season test of genotype differences against genotype-by-year variation. This check is reported only as a sensitivity analysis and not as a substitute for a properly specified random-year mixed model.

Drought-response indices were calculated from genotype mean grain yield under supplementary irrigation (Yp) and rainfed conditions (Ys): tolerance (TOL = Yp − Ys), mean productivity [MP = (Yp + Ys)/2], geometric mean productivity [GMP = (Yp × Ys)^0.5], yield stability index (YSI = Ys/Yp), stress susceptibility index [SSI = (1 − Ys/Yp)/SI, where SI = 1 − mean(Ys)/mean(Yp)], and stress tolerance index [STI = (Yp × Ys)/(mean Yp)^2] [48,49,50]. Because the supplementary-irrigated treatment was not demonstrably stress-free in the first season, these indices are interpreted only as treatment-based descriptive ranking metrics. No inferential significance test was applied to differences among index values. Indices were calculated separately by season and from pooled genotype means (Supplementary Table S4). Pearson correlations, PCA, and Ward clustering were based on genotype means for the 12 directly measured field traits after standardization to unit variance; pooled genotype means across the four environments were used for PCA and clustering, so these analyses describe overall phenotypic profiles rather than environment-independent genetic effects. Yield-derived indices were excluded from PCA and clustering. As sensitivity analyses, Spearman correlations of genotype yield ranks among environments and environment-specific Pearson correlations of grain yield with SPAD and heading time were calculated and are reported in Supplementary Table S10.

## 5. Conclusions

Across the two seasons and two water regimes, the strongest result was the magnitude of environmental dependence: the 2021–2022 rainfed environment approached crop failure, while genotype rankings were substantially more concordant between the two 2022–2023 environments. Fırat-93 showed the highest pooled STI/GMP ranking and high overall yield, whereas Şahinbey showed the highest pooled YSI but lower absolute productivity. These contrasts illustrate why productivity and proportional retention should be considered separately and why index rankings should not be treated as statistically distinct tolerance classes. Pooled SPAD, heading-time, and yield-component correlations were environment dependent and do not establish causal drought-tolerance mechanisms or validated selection criteria. The study is limited to two seasons at one site, with no stand counts, irrigation-depth record, plot-level soil-moisture data, or recoverable spatial randomization map. Future work should validate the candidate genotypes in multi-location, multi-year trials with fully quantified irrigation, water-balance measurements, stand establishment data, and mixed-model stability analyses before broad adaptation or breeding value is inferred.

## Figures and Tables

**Table 1:** F statistics from the combined ANOVA for 12 directly measured traits. Effect degrees of freedom are shown in the column headings; the complete ANOVA, including SS, MS, residual df (534), F, and *p* values, is provided in Supplementary Table S3. ** *p* < 0.01; * *p* < 0.05; ns, not significant.

Trait	Y (df = 1)	W (df = 1)	G (df = 44)	Y × W (df = 1)	Y × G (df = 44)	W × G (df = 44)	Y × W × G (df = 44)
Emergence time	9431.14 **	403.80 **	9.02 **	299.94 **	9.47 **	8.34 **	8.06 **
Heading time	151.21 **	19.31 **	36.24 **	129.34 **	17.04 **	1.98 **	2.21 **
Physiological maturity	875.86 **	1693.07 **	14.51 **	78.32 **	2.01 **	2.91 **	4.17 **
Heading SPAD	68.54 **	0.04 ns	9.90 **	80.22 **	1.64 **	1.53 *	1.95 **
Flowering SPAD	0.29 ns	9.30 **	9.00 **	5.10 *	3.60 **	1.49 *	0.96 ns
Thousand-kernel weight	809.09 **	3629.30 **	9.80 **	495.65 **	3.16 **	4.00 **	4.96 **
Grain yield	9542.46 **	20,360.29 **	90.98 **	1146.29 **	52.97 **	33.67 **	16.48 **
Plant height	8376.60 **	3744.11 **	32.70 **	1336.46 **	7.80 **	5.61 **	5.75 **
Spike length	1360.11 **	68.65 **	33.65 **	8.83 **	2.30 **	0.74 ns	1.63 **
Spikelets/spike	627.37 **	173.69 **	5.21 **	164.03 **	1.99 **	0.95 ns	1.08 ns
Grain weight/spike	470.40 **	1877.81 **	5.83 **	330.29 **	2.69 **	2.99 **	2.63 **
Grains/spike	597.83 **	1189.31 **	8.74 **	623.54 **	2.86 **	3.62 **	3.59 **

Notes: Y, growing season; W, water regime; G, genotype. ** *p* < 0.01; * *p* < 0.05; ns, not significant. The combined model treats the two observed seasons and water regimes as fixed environmental contrasts. Because the original spatial randomization map was not preserved and residual heterogeneity was detected, pooled-error F-tests should be interpreted cautiously; diagnostic results are reported in Supplementary Table S10.

**Table 2:** Mean trait values under rainfed and supplementary-irrigated conditions in the two growing seasons.

Trait	2021–22 R	2021–22 SI	2022–23 R	2022–23 SI
Heading (d)	151.93	148.85	152.12	153.48
Maturity (d)	182.57	187.86	185.97	194.16
Heading SPAD	49.43	51.48	53.28	51.33
Flowering SPAD	52.18	51.98	52.62	51.27
TKW (g)	32.61	43.48	34.38	58.01
Grain yield (kg ha^−1^)	466.5	4208.1	3254.3	5560.9
Height (cm)	52.23	89.61	101.20	110.62
Spike length (cm)	5.65	6.10	7.25	7.46
Spikelets/spike	14.20	17.80	19.44	19.49
Grain weight/spike (g)	0.47	1.50	1.14	1.56
Grains/spike	12.94	30.34	27.40	30.19

**Table 3:** Grain yield (kg ha^−1^) of 45 durum wheat genotypes across the four growing season × water-regime environments. Y1, 2021–2022; Y2, 2022–2023; R, rainfed; SI, supplementary irrigation. For readability, the main table reports numerical means only; the verified environment-specific Tukey HSD compact-letter groupings and pairwise-comparison interpretation are provided in Supplementary Table S9. The designation ‘43 IDSN 7135’ is the complete genotype name in the source records; ‘43’ is not a row number.

Genotype	Y1-R	Y1-SI	Y2-R	Y2-SI	Overall Mean
43 IDSN 7135	511.1	3655.0	2999.2	6014.7	3295.0
Anzele	515.3	4464.4	3789.4	6848.6	3904.4
Artuklu	379.9	4017.4	2148.9	3177.5	2430.9
AYM-3	355.8	6381.2	2566.7	4986.4	3572.5
AYM-6	379.8	4381.5	3690.6	6088.6	3635.1
Bağacak	559.7	6242.9	3528.5	6893.6	4306.2
Burgos	267.3	6418.6	4273.3	5986.4	4236.4
Cyprus-2	497.3	4490.4	3661.7	6684.4	3833.5
Diyarbakır-81	374.2	5294.9	3379.2	6731.2	3944.9
DYM-1	348.5	4059.2	3566.4	7397.9	3843.0
DYM-30	373.5	4140.9	3526.2	6054.0	3523.7
DYM-6	513.7	4260.6	2032.6	3259.7	2516.7
Edessa	536.1	3264.9	1995.1	2811.9	2152.0
Eyyubi	728.1	3165.4	2150.7	2659.9	2176.0
Fırat-93	502.9	5613.6	4371.9	6875.5	4341.0
Ganem	658.7	3777.5	3409.9	5623.1	3367.3
Gündaş	373.3	3250.3	2827.4	3186.5	2409.4
Güneyyıldızı	411.1	4286.6	4299.5	6876.9	3968.5
Hasanbey	492.2	4120.7	3702.5	5539.3	3463.7
Hat-286	360.9	4134.4	2469.6	3691.9	2664.2
Hat-301(B)	574.9	3068.4	2146.1	3527.2	2329.2
Havrani	551.0	3311.0	3025.1	3982.5	2717.4
Hevidi	642.7	3792.7	2253.2	4085.6	2693.5
Hundur	304.7	4076.4	3997.1	6368.3	3686.6
İskenderiye	402.6	3368.5	2445.3	4010.6	2556.7
Maestrale	507.2	2959.1	3627.9	6465.8	3390.0
MYM-12	282.6	3668.5	2015.6	2821.7	2197.1
MYM-8	358.9	3828.3	2638.6	2977.8	2450.9
NZFM-4	516.5	3877.5	3755.1	6183.2	3583.1
Ovidio	347.0	4180.2	3757.6	7292.6	3894.4
Seçkin-21	463.3	4639.8	3290.3	7441.0	3958.6
Selçuklu-97	595.0	4746.0	3229.7	7189.3	3940.0
Sena	569.5	4452.7	4004.9	7727.2	4188.6
Sham-1	466.4	5693.1	4017.9	6891.2	4267.1
Saragolla	548.0	3093.3	3379.2	6924.2	3486.2
Sorgül	401.7	3159.5	2867.1	6045.3	3118.4
Sümerli	393.6	5069.1	3451.4	7069.6	3995.9
Svevo	450.6	3986.0	3908.5	7001.8	3836.7
SYM-1	580.1	4391.5	2666.8	3307.9	2736.6
Şahinbey	383.8	3014.1	3981.8	5415.1	3198.7
Şampiyon	498.1	3269.6	3853.2	6180.8	3450.4
UYM-2	514.0	4183.8	2213.9	3698.5	2652.6
YM-16	503.1	4835.4	3810.3	6085.0	3808.5
YM-8	403.8	5497.9	3683.5	7211.2	4199.1
Zühre	595.6	3781.5	4034.6	6948.8	3840.1

**Table 4:** Pooled grain-yield drought-response indices. Yield-valued components are reported in kg ha^−1^. Yp, supplementary-irrigated yield; Ys, rainfed yield; TOL, tolerance; MP, mean productivity; GMP, geometric mean productivity; SSI, stress susceptibility index; STI, stress tolerance index; YSI, yield stability index. These pooled indices combine two seasons with markedly different stress intensities (SI = 0.889 in 2021–2022 and 0.415 in 2022–2023) and therefore must not be interpreted as rankings for the severe 2021–2022 rainfed environment alone; season-specific values are provided in Supplementary Table S4. Because YSI is a ratio (Ys/Yp), a high YSI can also occur in genotypes with modest absolute yield; YSI should therefore be interpreted together with an explicit productivity criterion.

Genotype	Yp (kg ha^−1^)	Ys (kg ha^−1^)	TOL (kg ha^−1^)	MP (kg ha^−1^)	GMP (kg ha^−1^)	SSI	STI	YSI
43 IDSN 7135	4834.8	1755.1	3079.7	3295.0	2913.0	1.029	0.356	0.363
Anzele	5656.5	2152.4	3504.1	3904.4	3489.3	1.001	0.510	0.381
Artuklu	3597.5	1264.4	2333.0	2430.9	2132.8	1.048	0.191	0.351
AYM-3	5683.8	1461.2	4222.6	3572.5	2881.9	1.200	0.348	0.257
AYM-6	5235.0	2035.2	3199.8	3635.1	3264.1	0.987	0.447	0.389
Bağacak	6568.2	2044.1	4524.1	4306.2	3664.2	1.113	0.563	0.311
Burgos	6202.5	2270.3	3932.2	4236.4	3752.5	1.024	0.590	0.366
Cyprus-2	5587.4	2079.5	3508.0	3833.5	3408.7	1.014	0.487	0.372
Diyarbakır-81	6013.1	1876.7	4136.4	3944.9	3359.3	1.111	0.473	0.312
DYM-1	5728.6	1957.4	3771.1	3843.0	3348.6	1.063	0.470	0.342
DYM-30	5097.5	1949.9	3147.6	3523.7	3152.7	0.997	0.417	0.383
DYM-6	3760.1	1273.2	2487.0	2516.7	2188.0	1.068	0.201	0.339
Edessa	3038.4	1265.6	1772.8	2152.0	1961.0	0.942	0.161	0.417
Eyyubi	2912.6	1439.4	1473.3	2176.0	2047.5	0.817	0.176	0.494
Fırat-93	6244.6	2437.4	3807.1	4341.0	3901.4	0.985	0.638	0.390
Ganem	4700.3	2034.3	2666.0	3367.3	3092.2	0.916	0.401	0.433
Gündaş	3218.4	1600.3	1618.1	2409.4	2269.5	0.812	0.216	0.497
Güneyyıldızı	5581.8	2355.3	3226.4	3968.5	3625.9	0.934	0.551	0.422
Hasanbey	4830.0	2097.4	2732.6	3463.7	3182.8	0.914	0.425	0.434
Hat-286	3913.2	1415.2	2497.9	2664.2	2353.3	1.031	0.232	0.362
Hat-301(B)	3297.8	1360.5	1937.3	2329.2	2118.2	0.949	0.188	0.413
Havrani	3646.8	1788.1	1858.7	2717.4	2553.5	0.823	0.273	0.490
Hevidi	3939.1	1447.9	2491.2	2693.5	2388.2	1.021	0.239	0.368
Hundur	5222.4	2150.9	3071.5	3686.6	3351.5	0.950	0.471	0.412
İskenderiye	3689.5	1423.9	2265.6	2556.7	2292.1	0.992	0.220	0.386
Maestrale	4712.5	2067.6	2644.9	3390.0	3121.4	0.907	0.408	0.439
MYM-12	3245.1	1149.1	2096.0	2197.1	1931.0	1.043	0.156	0.354
MYM-8	3403.0	1498.8	1904.3	2450.9	2258.4	0.904	0.214	0.440
NZFM-4	5030.4	2135.8	2894.5	3583.1	3277.8	0.929	0.450	0.425
Ovidio	5736.4	2052.3	3684.1	3894.4	3431.2	1.037	0.493	0.358
Seçkin-21	6040.4	1876.8	4163.6	3958.6	3367.0	1.113	0.475	0.311
Selçuklu-97	5967.6	1912.4	4055.3	3940.0	3378.2	1.098	0.478	0.320
Sena	6090.0	2287.2	3802.8	4188.6	3732.1	1.009	0.584	0.376
Sham-1	6292.2	2242.1	4050.0	4267.1	3756.0	1.040	0.591	0.356
Saragolla	5008.7	1963.6	3045.1	3486.2	3136.1	0.982	0.412	0.392
Sorgül	4602.4	1634.4	2968.0	3118.4	2742.6	1.042	0.315	0.355
Sümerli	6069.3	1922.5	4146.8	3995.9	3415.9	1.104	0.489	0.317
Svevo	5493.9	2179.5	3314.4	3836.7	3460.4	0.974	0.502	0.397
SYM-1	3849.7	1623.5	2226.2	2736.6	2500.0	0.934	0.262	0.422
Şahinbey	4214.6	2182.8	2031.8	3198.7	3033.1	0.779	0.386	0.518
Şampiyon	4725.2	2175.6	2549.6	3450.4	3206.3	0.872	0.431	0.460
UYM-2	3941.1	1364.0	2577.2	2652.6	2318.5	1.056	0.225	0.346
YM-16	5460.2	2156.7	3303.5	3808.5	3431.6	0.977	0.494	0.395
YM-8	6354.6	2043.6	4310.9	4199.1	3603.7	1.096	0.544	0.322
Zühre	5365.1	2315.1	3050.1	3840.1	3524.3	0.918	0.521	0.432

## Data Availability

The complete 720-observation replicate-level field dataset underlying the reported analyses is provided in Supplementary Table S8. Environment-specific summaries, drought-index calculations, PCA outputs, cluster membership, Tukey HSD comparisons, and reviewer-requested diagnostics/sensitivity analyses are provided in Supplementary Tables S2–S10. Additional File S1 supplies a reproducibility script that reads the replicate-level dataset and reproduces the principal reported outputs, including pooled drought indices, PCA explained variance and loadings, Ward cluster assignments, all environment-specific Tukey HSD pairwise comparisons with compact-letter verification, and the yield-rank sensitivity summaries. All reported numerical results were independently verified against the replicate-level dataset. Supplementary Table S8 is explicitly organized as genotype × environment × replication data (45 genotypes × 4 season × water-regime environments × 4 replications = 720 plot-level observations).

## Supplementary Materials

The following supporting information can be downloaded at: https://www.mdpi.com/article/10.3390/plants15213448/s1, Table S1: Identification and source/origin of the 45 durum wheat genotypes; Table S2: Genotype × growing season × water-regime means for all 12 directly measured traits; Table S3: Complete combined ANOVA; Table S4: Year-specific and pooled drought-response indices; Table S5: Pearson correlation matrix of directly measured field traits; Table S6: PCA explained variance, trait loadings, and genotype scores, including the PCA sign-convention note; Table S7: Ward cluster membership and the descriptive four-cluster-cut note; Table S8: Complete 720-observation replicate-level field dataset used for the publication analyses; Table S9: Environment-specific Tukey HSD genotype comparisons with verified compact-letter groupings; Table S10: Residual diagnostics, exact all-45-genotype yield-rank correlations, environment-specific correlations, Ward-cluster profiles, and second-revision verification notes. Additional File S1: Reproducibility Python script for drought indices, PCA loadings/scores, Ward cluster assignments, environment-specific Tukey HSD pairwise comparisons/letter verification, and the principal sensitivity analyses. All supplementary files listed here, including Additional File S1, are included in the final resubmission package. Table S8 now includes a subtitle that states this genotype × environment × replication structure explicitly.

## Abbreviations

The following abbreviations are used in this manuscript:

ANOVA	Analysis of variance
GMP	Geometric mean productivity
HSD	Honestly significant difference
MP	Mean productivity
PCA	Principal component analysis
SI	Stress intensity
SPAD	Soil Plant Analysis Development chlorophyll-meter reading
SSI	Stress susceptibility index
STI	Stress tolerance index
TKW	Thousand-kernel weight
TOL	Tolerance
Yp	Supplementary-irrigated genotype mean yield
Ys	Rainfed genotype mean yield
YSI	Yield stability index
